# Measuring vection: a review and critical evaluation of different methods for quantifying illusory self-motion

**DOI:** 10.3758/s13428-023-02148-8

**Published:** 2023-06-27

**Authors:** Lars Kooijman, Stefan Berti, Houshyar Asadi, Saeid Nahavandi, Behrang Keshavarz

**Affiliations:** 1 Institute for Intelligent Systems Research and Innovation, Deakin University, Geelong, Victoria Australia; 2 Institute of Psychology, Johannes Gutenberg-University Mainz, Mainz, Germany; 3 Harvard Paulson School of Engineering and Applied Sciences, Harvard University, Allston, MA 02134 USA; 4 Department of Psychology, Toronto Metropolitan University, Toronto, Ontario Canada; 5 KITE-Toronto Rehabilitation Institute, University Health Network (UHN), Toronto, Ontario Canada

**Keywords:** Self-motion, Measurement, Binary choice, Two-alternative forced choice, Magnitude estimation, Rating scales, Distance travelled, Chronometric

## Abstract

The sensation of self-motion in the absence of physical motion, known as *vection*, has been scientifically investigated for over a century. As objective measures of, or physiological correlates to, vection have yet to emerge, researchers have typically employed a variety of subjective methods to quantify the phenomenon of vection. These measures can be broadly categorized into the occurrence of vection (e.g., binary choice yes/no), temporal characteristics of vection (e.g., onset time/latency, duration), the quality of the vection experience (e.g., intensity rating scales, magnitude estimation), or indirect (e.g., distance travelled) measures. The present review provides an overview and critical evaluation of the most utilized vection measures to date and assesses their respective merit. Furthermore, recommendations for the selection of the most appropriate vection measures will be provided to assist with the process of vection research and to help improve the comparability of research findings across different vection studies.

## Introduction

The subjective experience of self-motion in the absence of actual physical motion is commonly termed *vection*. Vection is often exemplified by means of the “train illusion” (Kooijman et al., 2023). This illusion has been described to occur when a person is seated in a stationary train and another stationary train adjacent to the person starts moving. As a consequence, the person in the stationary train feels as if they are moving in the opposite direction of the adjacent train and perceive the adjacent train to be stationary (James, 1890). The first empirical documentation of the occurrence of vection due to visual stimulation goes back to work by Mach (1875). In one of his experiments (i.e., ‘*Versuch 1*’, p. 85-86), Mach described a rotating drum with equidistant vertical stripes (i.e., an *optokinetic drum*) that caused the observer to perceive illusory self-movement and the drum as stationary. Mach concluded that he felt a sensation of movement [“*Ich kann mich wenigstens eines Bewegungsgefühl nicht erwehren*” (p. 86)]. The first appearance of the actual term “*vection*” in the scientific literature can be traced back to work by Fischer and Wodak (1924), although Fischer and Kornmüller (1930) noted in their work that the term vection (i.e., ‘*Vektionen*’, p. 447), derived from the Latin verb ‘vehere’, was first coined by Tschermak in the early 1920s.

### The functional relevance of vection

Despite the long history of vection, research in this domain has recently gained more traction and attention. A literature search including the term “*vection*” (e.g., title, abstract, keywords) via different search engines (e.g., Scopus, Web of Science; 15 March 2023) revealed a total of 1076 articles published in this domain with a constant increase in vection-related research over the past years. The scientific scrutiny on vection is important for several reasons. Firstly, understanding how (illusory) self-motion perception is processed by our perceptual systems contributes to our knowledge of how humans perform functionally significant tasks in daily life. Palmisano et al. (2015) suggested that vection could be used to infer and control our actual self-motion, which is of importance when we navigate and spatially orientate ourselves. This functional role of vection is indicative from the research performed by Riecke et al. (2015), who showed that vection facilitates perspective switching, which is utilized in spatial orientation. Secondly, since vection may also tap into processing of actual self-motion, it allows researchers to investigate these self-motion processes when physical self-motion is not possible, for example when using complex neurophysiological imaging techniques such as functional magnetic resonance imaging (fMRI) (e.g., Kirollos et al., 2017; Kovács et al., 2008). Thirdly, understanding how motion perception occurs can be used to enhance the fidelity of virtual reality (VR) applications such as motion simulators (Hettinger et al., 2014). Previous research has shown that vection and presence (i.e., the feeling of “being there”, Heeter, 1992) are positively correlated (Riecke et al., 2005a), suggesting that vection is a desired sensation for VR applications. Lastly, vection has been associated with visually induced motion sickness (VIMS), a sensation similar to traditional motion sickness (Cha et al., 2021; Keshavarz & Golding, 2022). The relationship between vection and VIMS is rather complex (see Keshavarz et al., 2015b, for an overview) and mixed findings have been reported in the past (Kuiper et al., 2019; Nooij et al., 2017; Palmisano et al., 2007), highlighting the need for further research to better understand the relationship between vection and VIMS.

### Current challenges in vection research

Several conceptual and methodological concerns pervade the current vection literature. Firstly, there appears to be an inconsistency with regard to the definition of vection. Palmisano et al. (2015) screened 100 studies on how vection was defined and found that most studies described vection as a visually induced self-motion illusion. However, vection can be elicited through non-visual sensory modalities (see Hettinger et al., 2014, for an overview), including auditory (e.g., Väljamäe et al., 2005), biomechanical (e.g., Riecke et al., 2011), or tactile (e.g., Murovec et al., 2021) stimulation, making vection a rather multisensory phenomenon. There is a growing body of evidence that vection can be enhanced when several redundant sensory cues are simultaneously presented (e.g., Murovec et al., 2021; Riecke et al., 2011; Soave et al., 2020). Secondly, there is no consistency with regard to how vection is exemplified for participants in laboratory research studies. Vection is often verbally explained using the train illusion analogy (e.g., D’Amour et al., 2017; Ouarti et al., 2014; Stróżak et al., 2016, 2019; Tinga et al., 2018; Weech et al., 2020; Wright et al., 2006), but Soave et al. (2020) noted that this explanation did not appropriately reflect their participants’ experience of vection, which may alter the participants’ responses to the vection-inducing stimulation. Using practice trials to familiarize participants with vection is routinely applied, but there is no consistency with regard to the type of practice trial used (e.g., laboratory setting, stimulus).

Lastly, several researchers have pointed out the necessity for identifying objective measures to quantify vection (e.g., Keshavarz  et al., 2015a; Weech et al., 2020, see Palmisano et al., 2015 for a brief overview of objective measures). Promising approaches including the use of electroencephalography (e.g., Berti et al., 2019; McAssey et al., 2020) or postural measures (Weech et al., 2020) have been introduced recently; however, as these objective measures are still in their infancy and are not accessible to the broader research community, the vast majority of vection studies rely on subjective measures. In this regard, Väljamäe (2009) pointed out that vection research lacks a single and robust measure, and more than a decade later, this issue is still persistent in the literature; Berti and Keshavarz (2020) and Kooijman et al. (2022) both highlighted the variability in the use of vection measures in the context of neurophysiological and tactile-mediated vection studies, respectively. This variability in vection measures not only makes it increasingly difficult to interpret and compare results across vection studies, but also makes it challenging to understand the benefits and limitations of these measures and to choose the ones that are most appropriate for a respective research study.

### The present review

The goals of the present review are to (1) provide a general overview of the most common subjective measures used in vection research, (2) assess the merit of each measure, and (3) provide recommendations on their use for future vection research. Note that the aim of the present paper is not to offer an exhaustive overview of the vection literature per se; for this, we refer the reader to existing reviews for further discussions of vection and related factors (e.g., Berti & Keshavarz, 2020; Hettinger et al., 2014; Kooijman et al., 2022; Palmisano et al., 2015; Väljamäe, 2009). The current review does not review measurement techniques aimed to capture actual self-motion, as illusory self-motion (vection) and actual self-motion are two distinct concepts. The primary difference is the role of vestibular and proprioceptive feedback in actual self-motion (Britton & Arshad, 2019; Cullen & Zobeiri, 2021). The involvement of these sensory cues allows for measurement techniques that are unique to actual self-motion, such as estimations of heading (Cheng & Gu, 2018) or distance travelled (Harris et al., 2000), but do not directly capture vection. Furthermore, the review was neither designed as a systematic review, nor did it follow a meta-analytical approach to determine the efficacy of all the different vection measurement techniques that can be found in the current literature. The main reason for this is that the respective vection measure is typically not disclosed in the abstract or in the keywords, which makes a systematic/meta-analytical approach unfeasible. It is also important to note that the measures discussed in this review only focus on measures used to evaluate the immediate effect of vection-inducing stimulation, whereas vection aftereffects (e.g., see Seno et al., 2010; Seno et al., 2011) are not considered here. Lastly, we will focus solely on subjective measures and will not discuss the role of (neuro) physiological measures, such as electroencephalography (Keshavarz & Berti, 2014; McAssey et al., 2020; Palmisano, Barry, et al., 2016a), body sway (Mursic et al., 2017; Tanahashi et al., 2007), or fMRI (Kleinschmidt et al., 2002; Kovács et al., 2008), as these measures are not yet well-established and require further research.

## Measuring vection

**Fig. 1:**
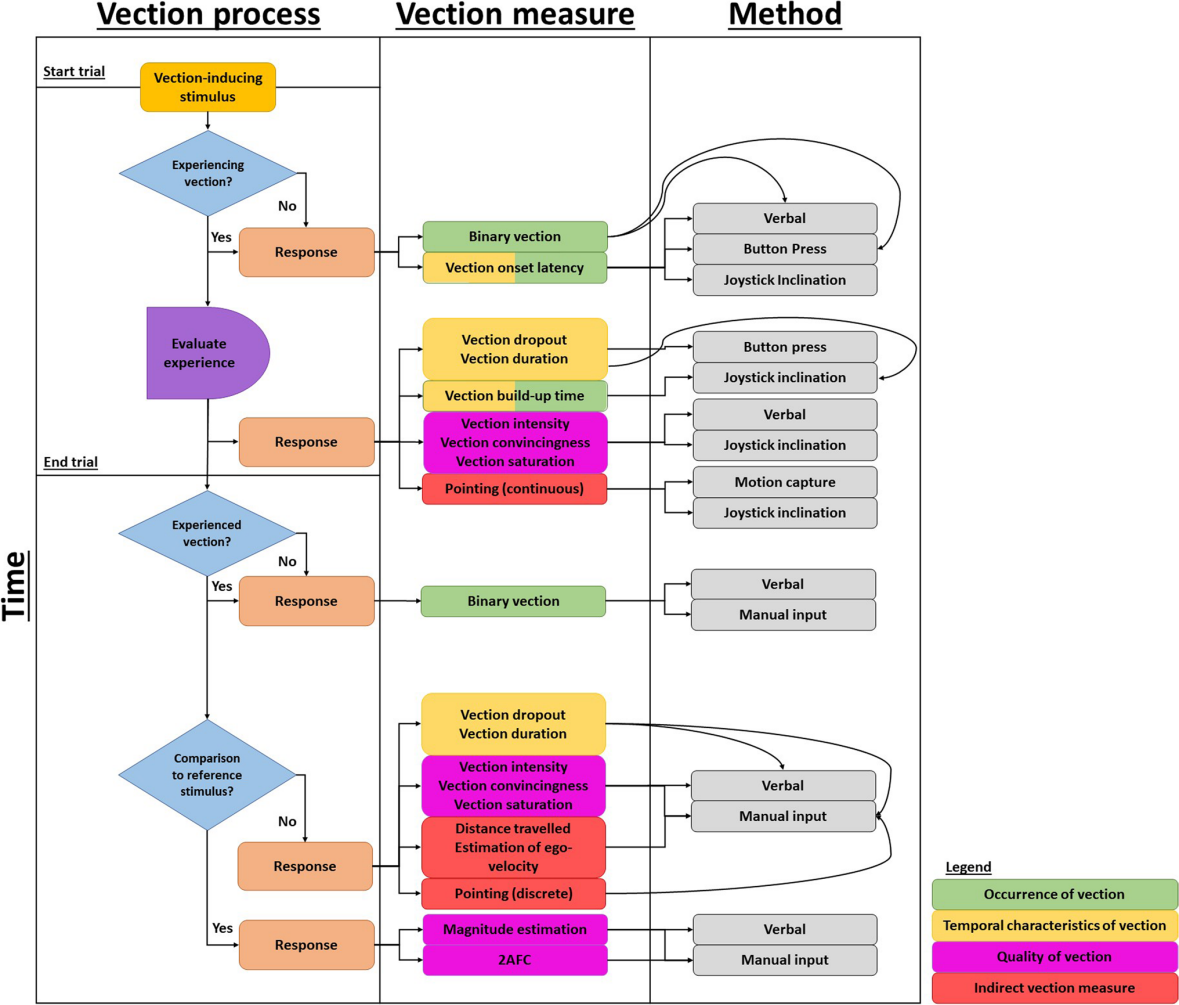
Vection measures used depending on when participants are probed during a vection experiment. *Note:* Measures can be either during the trial or post hoc, and can capture the occurrence of vection (green), the temporal characteristics of vection (yellow), or the quality of vection (magenta), or can indirectly capture vection (red)

A variety of techniques to quantify the experience of vection can be found in the literature. Here, we broadly separate them into four categories, namely measures that capture (1) the occurrence of vection, (2) the temporal characteristics of vection, and (3) the quality of the vection experience, and measures that (4) provide indirect estimations of the vection experience. It is important to note that the various measures can be applied at different points in time during an experimental vection trial, as depicted in Fig. 1.

The *occurrence of vection* can be captured after either stimulus onset or offset. That is, at the start of an experimental vection trial, measures evaluating the occurrence of vection could be utilized to determine whether participants experienced vection or not. Enquiring about the occurrence of vection can, for instance, be done via a “yes-no” choice during or after stimulus presentation (e.g., Kleinschmidt et al., 2002; Kovács et al., 2008). The *temporal characteristics of vection* are often assessed online during stimulus presentation and include measures of vection onset time/latency (e.g., Ouarti et al., 2014; Seya et al., 2015; Väljamäe et al., 2008), vection duration (e.g., Palmisano & Kim, 2009; Seno et al., 2018), vection build-up time (Riecke et al., 2005c, 2009b) and vection dropout (Guterman et al., 2012; Seno et al., 2018). It is also possible to assess vection duration and dropout after stimulus offset retrospectively. Note that vection onset time/latency can also inform about the occurrence of vection, making a specific binary choice obsolete if vection onset time/latency is measured. Similar to the temporal characteristics of vection, the *quality of the vection experience* (e.g., intensity/strength, convincingness/compellingness, saturation) can be measured during or after a vection trial using subjective rating scales (e.g., Allison et al., 1999; Berti et al., 2019; D’Amour et al., 2021; Kitazaki et al., 2019; Previc et al., 1993; Riecke et al., 2006). Upon cessation of the stimulus presentation, the quality of the vection experience can also be assessed using techniques that compare the vection experience to a previously presented stimulus. This can be done using a two-alternative forced choice approach (2AFC, e.g., Farkhatdinov et al., 2013; Ouarti et al., 2014) or magnitude estimation (e.g., Kirollos et al., 2017; Palmisano & Kim, 2009; Post, 1988; Seno et al., 2013). Lastly, *indirect measures*, such as estimations of travelled distance (Fauville et al., 2021; Nilsson et al., 2012; Nordahl et al., 2012; Wright et al., 2006) or pointing tasks (Lepecq et al., 1993; Riecke et al., 2015), have been introduced as potential measures that do not explicitly require a subjective estimation of vection and can be applied during or after stimulus presentation.

In sum, all of these measures have their benefits and limitations, making the choice of the appropriate measure rather challenging. In the following section, we will discuss the utility of each of the existing vection measures in depth, which will enable an educated choice when conducting vection research in general. Since these measures can be implemented at different stages during a vection experiment, they can be combined to capture multidimensional information on participants’ vection experience. A summary of the most common vection measures can also be found in Table 1.

**Table 1:** Overview of measures utilized in vection research (in alphabetical order)

	Category	Method	Example in Vection	Benefits	Limitations
Measure					
Binary response	Occurrence of vection	• Verbal • Button press	While experiencing a stimulus, participants indicate verbally or by pressing a button they are experiencing vection.	• Easy to understand by participants. • Allows categorizing trials as vection/no vection.	• Insensitive to quantify small differences (within-subject). • Requires large sample/repetitions.
Distance travelled	Indirect estimation of vection	• Verbal • Writing	After experiencing a stimulus, participants either indicate verbally or type in the number of meters they felt they had travelled.	• Gain insight of influence vection on (functional) self-motion processes.	• Does not measure *vection* directly. Thus, difficult to interpret. • Outcome potentially biased by (visual) context.
Estimation of ego-velocity	Indirect estimation of vection	• Verbal • Writing	After experiencing a stimulus, participants either indicate verbally or type in the velocity they felt at which they travelled.	• Gain insight of influence vection on (functional) self-motion processes.	• Does not measure *vection* directly. Thus, difficult to interpret. • Outcome potentially biased by (visual) context.
Magnitude estimation	The quality of the vection experience	• Joystick • Verbal • Writing	Participants experience a standard stimulus to which they ascribe an arbitrary value for their vection experience. Participants view subsequent stimuli and ascribe to these stimuli a value relative to the value they ascribed to the standard stimulus either verbally, via digital input or in writing to rate their vection experience.	• Sensitive to quantify small differences. • Able to account for individual differences.	• Between-subject variance in ratios requires data transformation. • Potential anchoring effect. • Limited cross-comparability between studies due to variability in methodology.
Pointing	Indirect estimation of vection	• Continuously point at target. • Point to remembered target at end of trial.	Participants either point continuously to a specific target while being subjected to a stimulus or point in the direction of a remembered target after experiencing a stimulus.	• Gain insight of influence vection on (functional) self-motion processes. • Online pointing reduces chance of memory-related artefacts.	• Does not measure *vection* directly. Thus, difficult to interpret.
Rating scales	The quality of the vection experience	• Joystick • Verbal • Writing	After experiencing a stimulus, participants rate their vection experience, such as the intensity or convincingness, based on a statement. The lower and upper ends of the rating scale have a description reflecting the extremes of participants’ vection experience.	• Sensitive to quantify small differences • Able to account for individual differences	• Terminology influences participants’ responses. • Scales with different intervals are not comparable
Two-alternative forced choice	The quality of the vection experience	• Verbal • Button press	After experiencing two stimuli, participants indicate which of the two stimuli elicited the strongest vection either verbally or by pressing a button (e.g., left or right mouse button).	• Less chance of response bias. • Easy to understand by participants.	• Requires large sample/repetitions.
Vection build-up time	Temporal characteristics of vection and the quality of the vection experience	• Joystick deflection	While experiencing a stimulus, participants move a joystick to indicate the intensity or convincingness of their vection experience. From the joystick data, the experimenter computes the time between vection onset and maximum vection.	• Allows for interpretation of multidimensionality of vection when complemented with other measures • Provides the option to correlate and interpretate (neuro)physiological measures.	• Potential to be affected by participants’ mental demands.
Vection dropout	Temporal characteristics of vection	• Verbal • Button press • Joystick deflection	While experiencing a stimulus, participants indicate the time they stop experiencing vection. From this data, the experimenter computes participants’ vection dropout.	• Provides the option to correlate and interpretate (neuro)physiological measures.	• No standard quantification of vection dropout exists. • Potential to be affected by participants’ mental demands.
Vection duration	Temporal characteristics of vection	• Verbal • Button press • Joystick deflection	While experiencing a stimulus, participants indicate the time they are experiencing vection. From this data, the experimenter computes participants’ vection duration.	• Allows for interpretation of multidimensionality of vection when complemented with other measures. • Provides the option to correlate and interpretate (neuro)physiological measures.	• Potential to be affected by participants’ mental demands.
Vection latency	Occurrence of vection and temporal characteristics of vection	• Verbal • Button press • Joystick deflection	While experiencing a stimulus, participants indicate verbally or by pressing a button they are experiencing vection and the experimenter records the moment in time relative to the start of the trial.	• Allows for interpretation of multidimensionality of vection when complemented with other measures. • Provides the option to correlate and interpretate (neuro)physiological measures.	Potential to be inflated by participants’ mental demands.

### The occurrence of vection

#### Binary choice

In vection research, binary choices are presented to participants by simply asking them whether they experienced vection or not. For example, participants in the study by Ohmi et al. (1987) were presented with a visual display aimed to induce circular vection and were asked to report the onset and cessation of circular vection. Similarly, participants in the studies conducted by Kleinschmidt et al. (2002) and Kovács et al. (2008) were exposed to a vection-inducing visual stimulus inside an MRI scanner and used buttons to indicate whether they perceived self-motion (i.e., vection) or object-motion. Please note that in some studies participants were presented with a vection-eliciting display and had to indicate in which direction they were experiencing vection (e.g., Larsson et al., 2004; Väljamäe et al., 2005). Although this might appear a binary option paradigm, it is in fact a ternary option paradigm since participants are able to indicate the direction of vection (i.e., left or right) as well as to indicate that they did not experience vection at all. Note that vection latency/onset time could also be regarded as a measure of vection occurrence since it measures the moment participants experience vection (see the section temporal characteristics of vection for details).

The binary response format is generally easy for participants to answer and takes less time to complete than a multi-category format (Dolnicar et al., 2011). Additionally, Dolnicar and Leisch (2012) showed that binary response formats were more stable, provided higher concurrent validity, and were completed faster than seven-point multi-category formats. As can be seen in Fig. 1, binary responses can be recorded at the earliest stage in the experimental trial. Thus, the binary response format can be used to abort trials upon a response (e.g., see Väljamäe et al., 2005) if one is merely interested in whether participants do or do not experience vection. However, the binary response format has several caveats. For instance, it suffers from the loss of information compared to multi-category formats (Dolnicar, 2003). Dichotomization treats individuals on opposing sides (yes/no) as different, whereas their responses could have been very similar to one another when measured using a continuous scale (Altman & Royston, 2006). Additionally, binary response formats require a large sample size to reach the same statistical power compared to continuous outcome variables (Bhandari et al., 2002). Lastly, a study by Bar-Hillel et al. (2014) showed a bias by participants presented with a binary choice, with the response option presented first being favoured by participants, which questions the validity of presenting participants with a binary choice.

### Temporal characteristics of vection

Time-related measures used in vection research revolve around the temporal characteristics of vection, such as the onset and duration of vection experienced by participants. The most common measures capturing the temporal characteristics of vection used in research are described in the following sections.

#### Vection latency/vection onset time

Vection latency (VL), also referred to as vection onset time, can be captured when participants verbally (Keshavarz et al., 2017; Väljamäe et al., 2008) or mechanically (e.g., button press: Howard & Howard, 1994; Ouarti et al., 2014; Palmisano & Chan, 2004; joystick deflection: Riecke et al., 2005b; Sauvan & Bonnet, 1993; Seya et al., 2015; mouse movement: Telford & Frost, 1993; potentiometers: Melcher & Henn, 1981) indicate the moment when they start to experience vection. Vection latency is computed by taking the difference between the moment a trial starts and the moment participants indicate they first experience vection, as shown in Eq. 1. It is worth noting that researchers might have different definitions of the start of a trial. For example, one might consider the start of the trial the moment when the visual cue first *appears*, whereas another might consider the start of the trial the moment the visual cue first starts *moving*. Presumably, the first account on the use of a VL measurement in vection research can be found in the study by Brandt et al. (1973). Participants in this study sat on a chair in an optokinetic drum which rotated around them, and the experimenter used a stopwatch to record the onset and offset of circular vection. In another study, Berthoz et al. (1975) derived VL from the position of a lever, which participants used to quantify the magnitude of the vection experience. Here, VL was derived from the moment the lever passed through a pre-defined threshold. Melcher and Henn (1981) recorded the onset of participants’ vection via a button press. Alternatively, Telford and Frost (1993) had participants move a computer mouse to indicate the moment and speed of their vection experience from which the authors determined VL. Sauvan and Bonnet (1993) instructed participants to indicate the onset of curvilinear vection by deflecting a joystick. Lastly, seated participants in a study by McAssey et al. (2020) viewed the projection of a rotating cloud of points on a dome-shaped surface and indicated the moment they started and stopped experiencing vection by pushing a button.

1 $$VL={t}_{vection, onset}-{t}_{trial, start}$$

The primary benefit of using VL is that it is easy to understand and to indicate by participants. Furthermore, it is easy for researchers to implement, either as a verbal measure or by letting participants press a button. Figure 1 shows that VL can be measured at the early stages of a trial and can function as a substitute or corroborator of the binary response measure. Furthermore, VL provides researchers with information on the temporal characteristics of vection that cannot be obtained by measures previously described. Recording VL allows us to clearly distinguish non-vection segments from vection segments. The segmentation is relevant, for instance, in (neuro)physiological studies that aim to compare (neuro)physiological responses during vection and non-vection episodes, which allows one to clearly identify the point in time during a trial when the perception from pure object-motion transitioned into a combination of object-motion and vection or pure vection. Lastly, when complemented with other measures, VL allows researchers to explore the temporal aspects of vection that typically coincide with vection intensity or convincingness but are yet distinct from them (e.g., see Seno et al., 2017).

One of the concerns surrounding the use of VL is that responses are likely to be delayed due to participants’ naivety, expectation, or confusion (Palmisano et al., 2015), and the comparability of VLs between studies can be impacted by the task instructions and definition of vection given by the experimenter. For example, participants may be asked to press the button as soon as they experience the slightest experience of vection in one study, whereas other studies might instruct participants to press a button once they are certain that they are experiencing vection. This ambiguity in task instruction may hamper the overall comparability of VL responses if they are not clearly stated in the respective publication.

#### Vection build-up time

Vection build-up time (VBT) is used as an indication of how long it takes for vection to reach a stimulus-dependent maximum. VBT is computed by taking the difference between the moment vection first occurs (i.e., VL) and the moment when the maximum reported vection occurs (Riecke et al., 2005b), as can be seen from Eq. 2. In studies conducted by Riecke and colleagues (e.g., Riecke et al., 2005b, 2005c, 2005d, 2009b), participants pulled a joystick in the direction they experienced circular vection and increased the angle of deflection proportional to the intensity of their vection experience. The point where the joystick reached its maximum angle was defined as maximum vection.

2 $$VBT={t}_{vection,\max }-{t}_{vection, onset}$$

Vection build-up time shares many of the benefits that have been listed for VL. That is, the recording of VBT is quite simple, time-efficient, and easy to understand for participants. In addition, when recorded together with other vection measures, they allow one to gain multidimensional information on participants’ vection experience. Again, VBT enables (neuro)physiological studies to specifically identify and focus on vection segments that contain the maximum vection the person could experience in relation to the stimulus, and compare them with pre- and post-vection segments. However, it is possible that strong and weak vection displays result in, on average, the same build-up time (e.g., see Figure 4 in Seya et al., 2015). The accuracy of VBT is impacted by the limitations presented for VL as the computation of VBT is dependent on the onset of vection. Additionally, the recording of VBTs requires the use of joysticks or sliders which requires the need for (1) training participants and (2) programming software to collect information on the position of the joystick/slider over time. As such, VBTs are mainly limited as joystick deflections and/or slider positions are prone to variability in ratings between participants.

#### Vection dropout

Vection dropout (VDO) describes the phenomenon when participants stop experiencing vection during an experimental trial (Young, 1991). Although VDO has been mentioned in the discussion or dissemination of various studies (e.g., Allison et al., 1999; Cheung & Howard, 1991; Palmisano et al., 2008; Seno et al., 2011), the measure has not commonly been reported on as a result in vection studies (Guterman et al., 2012; Seno et al., 2018). VDO can be quantified as (1) the number of times a participant stopped experiencing vection in a trial, (2) the proportion of trials with dropouts, (3) the latency until first dropout, (4) the average duration of dropouts, or (5) the total duration of dropout (see Guterman et al., 2012). Note that the total duration of dropout has been most commonly used as a VDO measure (Seno et al., 2012; Seno et al., 2018), which is summarized in Eq. 3.

3 $$VDO=\sum_{i=1}^n\left({t}_{vection, onset,i}-{t}_{vection, offset,i}\right)$$

The primary benefit of VDO is that it, akin to VL, offers information on the temporal characteristics of vection that other measures cannot offer, as VDO can be measured throughout an experimental trial (see Fig. 1), and thus provides researchers with the opportunity to clearly identify segments within a single trial where vection was not perceived. However, this does not hold true for post hoc measurements of VDO. The main limitation of VDO is the lack of a consistent definition of the measure (e.g., see Guterman et al., 2012 for various dropout measures). Furthermore, the reporting of VDO has potentially been made redundant by a vection duration measure expressed in percentage duration of a trial (Mursic & Palmisano, 2020), as described in the next section.

#### Vection duration

Vection duration (VD) is a measure reflecting how long participants’ vection experience lasted and is expressed either in seconds (Kirollos & Herdman, 2021; Palmisano & Kim, 2009; Weech et al., 2020) or in percentage of total trial duration (D’Amour et al., 2017; Seno et al., 2018). Various techniques have been employed to record vection duration. For example, Gurnsey et al. (1998) had participants press a button when they experienced vection during the presentation of the stimulus from which the authors derived vection duration. Similarly, Kirollos and Herdman (2021) asked participants to press and hold a button on a controller while they experienced vection, which was used to calculate VD. Palmisano and Kim (2009) and Weech et al. (2020) used a similar approach, only with different pieces of hardware (e.g., joystick or mouse button press, respectively). Participants in the study conducted by Keshavarz et al. (2017) estimated the duration of their vection experience after the stimulus presentation had ceased.

Despite VD being a commonly employed measure, it is often not explicitly mentioned how VD is computed, and inferences must be made from the procedural descriptions in the manuscripts. The appropriate method to determine VD would be either (1) by taking the sum of differences between segments wherein vection onset and dropout occur (Eq. 4), or (2) by dividing Eq. 4 by the total duration of the trial (Eq. 5). The latter approach was used by Seno et al. (2018) to derive VD and to account for varying trial durations. Alternatively, some researchers asked participants to verbally report the duration of vection in percentages post hoc (D’Amour et al., 2017; Keshavarz et al., 2017; Murovec et al., 2021), where 0% indicated that participants experienced no vection at all and 100% indicated that they experienced vection throughout the trial.

4 $$VD=\sum\nolimits_{i=1}^n\left({t}_{vection, dropout,i}-{t}_{vection, onset,i}\right)$$

5 $$VD=\frac{\sum_{i=1}^n\left({t}_{vection, dropout,i}-{t}_{vection, onset,i}\right)}{t_{duration, trial}}$$

Similar to VL, the main benefit of recording VD is that it is easy to understand and to indicate by participants and to implement by researchers, either as a verbal measure or by letting participants press a button. Akin to VL, VBT, and VDO, VD offers information on the temporal characteristics of vection that other measures cannot offer. Figure 1 shows that VD can be measured throughout the trial, and it thus provides researchers with the opportunity to clearly identify segments within a single trial where vection was perceived. However, this holds true only when VD is assessed using, for example, a button press, but not with post hoc verbal assessments. The segmentation of vection trials can be helpful for (neuro)physiological studies, where (neuro)physiological responses can be interpreted based on VDs, allowing one to compare stages pre-vection, during vection, and post-vection. Lastly, complementing VD with other measures can provide researchers a broader picture on participants’ subjective experience. For example, prolonged VDs might coincide with reduced VL and increased vection intensity. Indeed, the study by Seno et al. (2017) showed that generally longer VDs correlated with shorter VLs and higher vection intensities. Furthermore, the model developed by the authors, which used VD, VL, and vection magnitude as indices, was able to predict participants’ vection experience to a reasonable degree. Nonetheless, with the potential variability in which VD could be calculated, and the lack of reporting on the way VD is calculated, the comparability of research findings is hampered. Moreover, the accuracy of VDs is impacted by the limitations presented for VLs as the computation of VD is dependent on the onset of vection.

### The quality of the vection experience

Measures that evaluate the quality of the vection experience revolve around measuring aspects such as the intensity/strength or convincingness/compellingness of the vection experience. These measures either can be discrete or can make use of a comparison to previously shown standard stimuli. The most commonly used measures to assess the quality of the vection experience are described in the following sections.

#### Two-alternative forced choice (2AFC) task

The 2AFC task is an elementary method to measure the sensitivity of participants to sensory input and is commonly used to determine human perceptual thresholds (e.g., see Camacho et al., 2015; Wang et al., 2016) or the point of subjective equality (Ulrich & Vorbergb, 2013). Although the 2AFC method has not been used extensively in vection research, its use can be exemplified through the experiments by Farkhatdinov et al. (2013) and Ouarti et al. (2014). Participants in the experiment by Farkhatdinov et al. (2013) were exposed to a sequence of two visual-vibrotactile stimuli. The speed of the visual stimulus was constant over all trials, whereas the intensity and frequency of the vibrotactile stimulation were different between the pairs. Participants then indicated which of the two stimuli elicited stronger vection. Similarly, Ouarti et al. (2014) presented participants with a visual scene showing a cart moving through a tunnel while haptic feedback was provided by asking participants to hold onto a handle. The handle moved in proportion to the acceleration of the virtual cart. Following the 2AFC paradigm, participants were presented with two sequential trials with unique combinations of visual and haptic feedback and indicated which trial elicited stronger vection.

The practical benefit of employing a 2AFC task is that it is easy to understand, simple for participants to perform, and typically not prone to response biases (Peters et al., 2016). However, similar to binary measures, the dichotomization of outcomes comes at a cost of loss of information and requires a large sample size or many repetitions. In the context of vection, the most apparent limitation of a 2AFC paradigm is that there is no option for participants to disclose that they did not perceive vection at all. That is, participants are forced to choose the stimulus that generated stronger vection, even if neither of the two sequentially presented stimuli elicited vection at all. Thus, there is a risk that participants base their decision on simple heuristics, such as visual velocity or vibrational intensity, instead of the actual experience of vection. To counteract this, adding a “no” option can be considered (Dhar & Simonson, 2003). Figure 1 shows that 2AFC paradigms are mostly employed at the end stage of a trial. As such, this method is prone to memory-related artefacts; upon presentation of the standard stimulus, participants have to retain the vection information of the standard stimulus during the presentation of the subsequent stimulus, evaluate their vection during the second stimulus, and compare the vection experienced during the standard to the vection experienced during the subsequent stimulus. Thus, this method can be cognitively complex and affect participants’ performance in accurately reporting on their vection experience. Lastly, as 2AFC paradigms involve the sequential presentation of two stimuli, multiple trials/repetitions are necessary and thus stimulus durations are often kept relatively short. For example, in the study by Farkhatdinov et al. (2013), 36 pairs of stimuli were presented, with each stimulus lasting 10 seconds, whereas Ouarti et al. (2014) presented 24 pairs of stimuli each having a duration of 25 seconds. However, vection typically takes up to 10 seconds to occur (Berthoz et al., 1975; Palmisano & Riecke, 2018), and thus vection-inducing stimuli are often of longer duration. In return, longer stimulus durations can be problematic by increasing the risk of memory-related artefacts.

#### Magnitude estimation

The paradigm of magnitude estimation (ME) was introduced by Stevens (1956, 1957) in the context of psychophysics and was used to obtain judgements from participants on the perceived intensity of a certain stimulus with respect to a predetermined standard stimulus. In the original form of ME, participants are first presented with a standard stimulus, such as a sound, to which an arbitrary number, such as *50* (i.e., the modulus), is ascribed. When presented with subsequent stimuli, participants must rate the perceived intensity of these subsequent stimuli with respect to the standard stimulus. According to Stevens, this procedure allows one to identify a power law that describes the relationship between the physical increase of a stimulus and the perceived change in stimulus intensity. However, Stevens later argued for the abandonment of presenting a modulus stimulus (Stevens & Marks, 2017).

The implementation of ME in vection research can be exemplified through a study by Berthoz et al. (1975), where participants were shown a visual stimulus moving at 1 m/s and were instructed that vection experienced during this stimulus should be rated as 100%; this stimulus was considered the standard. To explain the procedure of ME to the participants, the authors then presented a subsequent stimulus moving at a velocity of 0.5 m/s and participants were instructed that vection experienced during this stimulus should be rated as 50%. During the subsequent experimental trials, participants used a lever to indicate the magnitude of their vection experience for each trial with respect to the standard. Brandt et al. (1971) presented participants with a standard stimulus during which both the participant and the optokinetic drum rotated at 60 degrees per second. Participants were instructed to ascribe the value 6 to their experience and estimate subsequent stimuli relative to this experience. Participants in the study by Post (1988) were subjected to vection-inducing stimuli and utilized magnitude estimation to report on circular vection within a range of 0 to 10, where 0 represented the sensation of stationarity and 10 represented the vection experience during a prior stimulation. In another study by Kirollos and Herdman (2021), participants viewed a pattern of vertical stripes rotating around the yaw axis through a head-mounted display. At the start of the experiment, participants were exposed to a visual scene that consisted of vertical stripes rotating clockwise and counterclockwise for 20 s each. A value of ‘50’ in terms of vection intensity was ascribed to this stimulus (i.e., the standard stimulus), and participants rated subsequent stimuli on a 0 to 100 scale with reference to this standard. Similarly, Palmisano et al. (2016b) presented participants with a standard stimulus at the start of each block of experimental trials and instructed participants that if they felt they were moving during the standard stimulus it corresponded to a value of 5. In subsequent trials, participants rated the vection intensity after viewing each display by changing the size of a bar chart that had a range from 0 to 10.

The primary benefit of ME is that it provides information on how changes in the physical property of a stimulus (e.g., speed) influence participants’ experience of vection (e.g., intensity). Furthermore, ME provides researchers with ratios that have a greater sensitivity to measuring small differences compared with categorical scales (Grant et al., 1990). However, some researchers who have employed ME in vection research have utilized Stevens’ original method wherein a numerical value was prescribed for the modulus and defined ranges from wherein participants could choose numbers. The prescription of a numerical value for the modulus could have presented anchoring as an issue in this regard (see Furnham & Boo, 2011, for a review on anchoring). For example, the presence of an anchor, whether physical or numerical, appears to influence participants’ ability to estimate the length of a line (LeBoeuf & Shafir, 2006). Thus, presenting participants with a numerical value for the modulus vection stimulus may anchor the range of numbers from which participants sample their responses. For example, if the number ‘5’ was ascribed to the modulus stimulus, participants may likely respond with (whole) numbers that do not deviate much from 5, whereas if the number 50 was ascribed to the modulus, participants may likely respond with whole numbers that do not deviate much from 50. In relation to vection research, ME suffers from a similar limitation as the 2AFC paradigm and is prone to memory-related artefacts; participants must retain their vection experience during the standard stimulus and compare it to the subsequent stimulus, which can be cognitively cumbersome and could introduce memory-related artefacts (Carpenter-Smith et al., 1995). Another limitation of the use of ME in vection research is that, when utilizing the original method where a modulus stimulus is employed, the strength of the stimulus is not defined unambiguously (Carpenter-Smith & Parker, 1992). The degree to which participants experience vection during the presentation of the standard stimulus may differ inter-individually, and thus the ratio between the standard and the subsequent ratings may also differ between participants. For instance, participants are typically asked to assign a certain number to a vection-inducing standard stimulus (Palmisano & Kim, 2009; Weech et al., 2020), although the standard stimulus may in fact induce strong vection in some participants and no vection in others, in which case they should be excluded from the dataset. Thus, the standard stimulus cannot be considered a robust standard that is equal across all participants, unlike physical units such as weight, loudness, or length. As a result, ME can (1) only inform about changes in vection ratings, and does not allow one to draw any conclusions on the absolute intensity of an individual’s vection experience and (2) result in substantial loss in data if participants fail to experience vection during the standard stimulus. Additionally, there is a considerable variability in how ME is applied, and therefore cross-comparability of research findings across studies is difficult (Miller et al., 2015). Similarly, the characteristics of the standard and the subsequent stimuli vary between studies, further complicating the comparability of research findings.

#### Rating scales

Participants are typically instructed to utilize rating scales to rate (1) the ‘*intensity*’ of their vection experience, (2) the ‘*convincingness*’ of their vection experience, (3) both the ‘*intensity*’ and ‘*convincingness*’ or (4) the degree of vection saturation. The measurement of vection intensity can be exemplified using the study by Previc et al. (1993). Participants in this study rated their vection on a five-point scale where *1 = little or none, 2 = below average, 3 = average, 4 = above average, and 5 = a great deal of vection.* Participants in the study by D’Amour et al. (2021) viewed alternating black-and-white horizontal bars inducing circular vection and were asked to rate vection intensity (“*How strong was the sensation of vection*?”) on a scale ranging from 0 (*no vection*) to 10 (*very strong vection*). Similarly, Kitazaki et al. (2019) measured vection intensity using a visual analogue scale (VAS) (“*I felt that my whole body was moving forward*”) in participants who were presented with first-person perspective recordings of someone walking. Riecke et al. (2011) exposed participants to vection-inducing auditory cues while participants performed side-stepping motions on a circular treadmill. The researchers in this study asked participants to verbally indicate vection intensity (“*How intense was the sensation of self-motion on a scale between 0 and 100%?”). *Please note, vection intensity is sometimes referred to as vection strength; however, herein we adhere to the term vection intensity.

An example of vection convincingness measures can be found in a study by Lind et al. (2016), where participants laid on an actuated wooden platform and were exposed to a visual scene using VR glasses that suggested sandboarding down a dune. After each trial, participants completed a series of questions, one of which asked participants to rate the convincingness of the sensation of movement using a 0-to-100 scale. In another study, Riecke et al. (2006) presented participants with rotating 360-degree images of a market environment which were scrambled to various degrees. After trial completion, participants used a joystick to rate the convincingness of vection on a scale ranging from 0% (“*no perceived motion at all*”) to 100% (“*very convincing sense of vection*”). Please note, vection convincingness is sometimes referred to as vection compellingness or vectionrealism, but herein we adhere to the term vection convincingness.

Vection saturation was recorded in the study by Allison et al. (1999), where participants sat on a stationary chair placed in a furnished room that could rotate around the participants’ roll axis. After each trial, participants reported their perceived velocity relative to the velocity of the tumbling room on a seven-point scale, where “0” reflected those participants only perceived the room to be moving and “6” reflected that participants only perceived themselves to be moving. Similarly, Guterman and Allison (2019) utilized a rating of vection saturation in their study where participants sat or laid on a foam mattress while looking at a display that aimed to elicit vection. However, the saturation rating scale in this study ranged from 0 to 100, where 0 reflected that the scene was perceived to be moving and the participant felt they were stationary and 100 reflected that the scene was stationary and the participant felt they were moving.

The main benefit of using rating scales is that they allow one to easily capture complex human behaviour (Parker et al., 2013) or multiple health states at the same time (Bleichrodt & Johannesson, 1997); by providing participants with multiple statements to rate, researchers can identify and disentangle different behaviours or health states which might co-occur. As such, rating scales offer more variability in response options compared with, for example, binary choice options. Another benefit of rating scales is that they can be employed either during the trial or directly upon trial completion (see Fig. 1), which decreases the chance of memory-related artefacts occurring. Furthermore, rating scales are generally easy to implement by researchers and easy to understand and to use by participants. However, some caveats exist for the use of rating scales. Parker et al. (2013) investigated the reliability of dichotomous and multicategory scales by deriving six different gradations (i.e., 2-, 3-, 5-, 7-, 10-, and 15-item points) from a quasi-continuous dataset. Their results showed that the performance of scale reliability indices (e.g., Pearson’s *r*, Cramer’s *V*) behaved differently for each gradation and appeared to be scale-dependent. Moreover, reliability indices did not remain constant when a higher number of gradations were collapsed into fewer. As such, there is limited comparability between studies using scales with different gradients. Similarly, it is problematic for comparability across studies if vection rating scales are used in different ways. For example, a rating scale can range from *0* to *10*, with “zero” representing no vection. However, the same scale can be used as a follow-up question after a participant has already indicated having experienced vection, where the scale value “zero” becomes de facto redundant and could be omitted. This raises the question whether the two scales depict the same measure in both cases and are still comparable or whether participants scale their experience differently in the two cases.

### Indirect vection measures

Besides asking participants about their vection experience directly, participants’ vection experience might also be assessed through indirect measures. Some of these measures offer participants the opportunity to quantify their vection experience in terms of physical motion properties, such as estimations of the distance travelled (Fauville et al., 2021; Nilsson et al., 2012; Nordahl et al., 2012; Wright et al., 2006) or estimations of self-motion velocity (Palmisano et al., 2006; Riecke et al., 2009a). Other measures attempt to quantify participants’ vection experience in terms of spatial orientation (e.g., Lepecq et al., 1993). However, these measures do not directly measure vection in the sense that they attempt to quantify vection intensity, convincingness, saturation, onset, or duration, but rather they serve as an indicator of the perception of vection. Note that accurate performance in some of these indirect measures (e.g., estimation of self-motion velocity) can be achieved based on visual parameters alone (e.g., optic flow) and does not necessitate the ability to experience vection, questioning the validity of such measures for vection research. However, the implementation of indirect measures allows for a more direct investigation of the functional significance of vection (i.e., the effect of self-motion on behavioural adaptation). Thus, we will briefly discuss the employment of a few indirect measures which are commonly used and highlight their individual benefits and limitations.

#### Pointing

The pointing technique requires participants to either point to a remembered target (Lepecq et al., 1993) or continuously point towards the perceived location of a target (Riecke et al., 2015). Pointing tasks are predominantly performed with participants having their eyes closed (e.g., Riecke et al., 2015; Siegle et al., 2009). Lepecq et al. (1993) hypothesized that if participants experienced vection, their pointing angle would deviate from the actual position of a remembered target prior to the vection experience. To test this, participants performed three different pointing tasks, namely (1) pointing to visually present targets, (2) pointing to the memorized direction of previously presented visual targets, and (3) pointing to the memorized direction of previously presented visual targets after viewing a display aimed to elicit forward vection. The authors found that the pointing error increased when participants pointed to targets in their lateral field of view after being exposed to a vection-inducing display. In a study by Riecke et al. (2015), blindfolded participants were seated in a hammock chair and used a joystick to continuously point to the location of the sound of an owl that surrounded them. The authors hypothesized that if participants truly experienced vection, they would change their pointing direction to follow the illusory motion of the auditory cue. Depending on the condition, stereo or mono recordings of the rotating sound field were presented to participants to account for the possibility of external sounds influencing their perception. No significant differences in pointing errors were found between conditions in which participants experienced vection and conditions in which participants physically moved.

The primary benefit of pointing measures is their ability to quantify participants’ vection experience in the form of a physical motion property that does not rely on subjective ratings. These physical motion properties could be related to functional motion processes. For example, the study by Riecke et al. (2015) showed that the experience of vection can influence participants’ pointing error and facilitate perspective switches, thereby indicating that vection can affect functional processes. As can be seen from Fig. 1, pointing can be employed as a continuous measure, which reduces the possibility of memory-related artefacts during post hoc judgements and can capture online self-motion processing (Siegle et al., 2009). However, the major limitation of pointing tasks is that the interpretation of which pointing error metric mirrors (potential) changes in vection is difficult. For example, it is not clear whether a larger deviation in pointing angle to a remembered target truly indicates a stronger vection experience or whether this deviation is a result of stimulus context/characteristics. As such, pointing tasks could be used to complement more ‘conventional’ vection measures. Another limitation is that pointing tasks require certain equipment that allows one to accurately measure certain body movements, and this equipment might not be easily accessible.

#### Estimation of physical motion properties

*Distance travelled*. Generally, the distance travelled (DT) measure describes how far participants perceived they moved (or travelled) during an immersive virtual scene. For instance, participants in the study conducted by Harris et al. (2000) were presented with a depiction of a virtual corridor. Participants were either (1) physically moved or (2) only shown visual motion, or (3) were subjected to a combination of both. Participants pressed a button when they perceived they moved through a target in the corridor, which was converted by the researchers into a perceived distance travelled. Participants in a study by Nilsson et al. (2012) stood on a platform and were exposed to four different static VR scenes while being subjected to vibrations to their feet eliciting haptically induced vection. After each trial, participants estimated the distance they had virtually travelled in meters for each of the different VR scenes. The authors used DT in their study as an indication of vection intensity while they also verbally collected vection convincingness ratings. Similarly, in Nordahl et al. (2012), participants who were standing on a platform were visually immersed in a virtual elevator; again, participants estimated the vertical distance they had virtually travelled in meters. In another study, Fauville et al. (2021) showed participants an orange marker located either on the floor or in the water of an actual swimming pool prior to immersing participants in a virtual environment wherein they perceived themselves to be swimming. Upon completion of a swimming trial, participants were asked to indicate how far they had travelled from the orange marker.

*Estimation of ego-velocity*. Similarly to DT, the estimation of ego-velocity (EEV) measure describes participants’ vection experience in terms of a physical motion property. However, instead of distance, participants indicate how fast they perceived themselves to be moving. For example, Post (1988) had participants in their vection study rate their perceived velocity using magnitude estimation where *0* represented stationarity and *10* represented the perceived velocity during a prior standard stimulation. Alternatively, Telford and Frost (1993) had participants continuously move a computer mouse in front of them at the speed at which the participants perceived to be moving while they were subjected to vection-inducing stimuli. Participants in a study by Palmisano et al. (2006) sat on a stationary chair located in a tumbling room that could rotate around participants’ roll axis. After experiencing the rotating room for 30 seconds, participants were asked to indicate how fast they felt they were moving relative to a standard stimulus of 10°/s using a magnitude estimation paradigm. In another study by Riecke et al. (2009b), blindfolded participants were presented with auditory targets in a rotating sound field and were asked to call out the name of the auditory target when they believed they were facing the target. In this study, self-motion velocity was estimated by the authors by dividing the total turned angle by the total duration of vection experienced by participants in the trial. Furthermore, speed matching and nulling tasks have also been employed in vection research. An example of speed matching can be found in the study by Kim and Palmisano (2008), where participants were presented with a variety of visual displays simulating forward or backward self-motion, and each display was succeeded by a rating display. During this rating display, participants were asked to dynamically change the velocity of the rating display to match the perceived velocity of the previous stimulus. An example of speed nulling can be found in the study by Palmisano and Gillam (1998), where participants sat in an optokinetic drum which rotated around them. Subsequently, participants were instructed to change the rotational speed of the chair they were sitting on in the same direction as the rotation of the drum until they did not experience vection anymore (i.e., until participants perceived they were stationary).

The main benefit of DT and EEV is that participants can quantify their vection experience through a metric of length (e.g., metres, feet, or yards) or speed (e.g., degrees/s or m/s). However, as with the pointing paradigm, it is currently unclear how DT and EEV are to be interpreted in relation to vection. For example, it is unclear how changes in DT and EEV reflect actual changes in vection perception. That is, it remains uncertain whether a larger DT or EEV indicates a more intense or more convincing vection experience. For instance, previous research by Bremmer and Lappe (1999) showed that participants can utilize visual information alone to accurately reproduce DT estimations. As such, it is possible that participants rely on visual information to make DT judgements rather than deriving this estimate from their vection experience. Furthermore, Nilsson et al. (2012) argued that the larger DT found in one of their four experimental conditions does not necessarily imply that vection was “*superior to the ones elicited by (…) the other two conditions for that matter,*” (p. 358) as vection convincingness ratings did not differ between conditions. Instead, participants’ DT estimation could have been affected by the context of the virtual scene according to the authors. Lastly, the utility of DT is limited to studies on *linear vection* as it cannot be applied in its current form to studies on *circular vection*. Nonetheless, DT and/or EEV could be used as a complementary measure.

## Discussion and recommendations

The summary of the existing measures applied in vection research demonstrates the substantial heterogeneity in methods used to capture vection. Furthermore, it shows the lack of established methodological procedures that are generally agreed upon in the research community. This lack of established methodological procedures raises the question of how the multitude of vection measures, which are all supposed to capture the same phenomenon, should be evaluated. The multitude of measures indicates two things: Firstly, the variety in vection measures mirrors the complexity of quantifying the phenomenon on an individual basis and, secondly, the multitude of measures utilized in vection research impairs the comparability and integration of studies and their findings.

Vection can be perceived in very different ways. For example, the same visual input may generate a strong sense of vection that lasts only a short period of time in one observer, while another observer may experience only a faint experience of vection that starts very quickly and lasts for a prolonged time. A third observer, in contrast, may experience no vection at all. From this perspective, it seems beneficial for researchers to have a broad variety of measurement tools available to capture the different aspects of vection. However, the potential variance in participants’ vection experience also implies that a single measure that could fit all situations does not exist, and that the appropriate measures need to be carefully selected on an individual (i.e., experimental level) basis. Such a selection requires thorough consideration of at least two aspects when designing and conducting an experimental study: (a) the general research question and (b) the specific characteristics of vection that best represent the research question. For instance, imaging studies investigating the neurophysiological correlates of vection may only be interested in comparing vection versus non-vection episodes, whereas individual differences in vection duration and/or intensity might not be relevant. In such a case, it seems reasonable to choose a binary (yes/no) response format (e.g., using a button press) to accurately differentiate between vection and non-vection episodes. In contrast, studies exploring the influence of cognitive aspects on vection may very well be interested in nuanced differences in vection perception, making the choice of vection intensity, duration, and onset measures appropriate. Thus, it seems generally a good idea to apply several different measurement methods when appropriate rather than just one. But, of course, pragmatic limitations regarding the number of measures that can be applied during an experiment also need to be considered.

The multitude of measures utilized in vection research impairs the comparability and integration of studies and their findings. It is particularly problematic if individual studies not only use different measures in principle, but if these measures are also used in different fashion. The main issue overall is that the reasons behind the choice of the specific measures and settings are rarely communicated in the dissemination of the results. To allow for comparability of studies, or to be able to evaluate the incomparability of individual studies, greater transparency is needed: the exact settings used in each measure, but also all details of instruction and (vection-inducing) stimulation should always be reported. Unfortunately, this accuracy is not consistently met (see Berti & Keshavarz, 2020, for further information). Additionally, the lack of transparency does not afford researchers insight into the terminology used to query vection. One approach to achieve comparability between measures would be to investigate how participants experience vection and what wording should be used to query and describe their experience (e.g., see Soave et al., 2021). Such qualitative research could identify appropriate terminology that should be used to (1) define vection, (2) exemplify the concept of vection to participants, and (3) determine how to formulate vection measures, such as rating scales.

In sum, the following recommendations seem appropriate to us for guiding the selection of one or more vection measures for a specific study:

1. As the first step in choosing the appropriate vection measures, it is important to be aware of the different definitions and types of vection, which can be found in the empirical literature (see Palmisano et al., 2015). Based on this, it is important to explicitly set the relevant vection definition for the study. The selected definition may already limit the applicable vection measures and can, for example, assist in defining how chronometric measures are computed.

2. One could consider combining measures (e.g., ratings scales for intensity/convincingness and temporal characteristics measures, such as VD or VDO) to capture the different aspects of vection. Combining measures would allow for the application of advanced  statistical analysis of the data to test the complex experience of vection in a more holistic way (e.g., see Seno et al., 2017).

3. Based on the task that participants are expected to perform, a preliminary selection of the type of vection measure could be made. For example, if participants are required to use their hands to control a steering wheel, measures derived from joysticks or button presses might not be a convenient option and one might have to resort to verbal measures. Furthermore, a trade-off must be made between experimental demands and memory-related artefacts. Online measures, such as button presses or joystick inclinations, might increase the experimental demands imposed on the participant while avoiding memory-related artefacts in a measure. However, if participants are expected to perform multiple tasks during a trial, it might be beneficial to initiate some measurements *after* the trial to reduce experimental demands *during* the trial. For example, one could implement a button press during the trial to gain insight on vection onset and duration, but measure vection intensity and/or convincingness after completing the trial. Additionally, caution should be exerted in the number of measures used; as it is recommended to measure vection, presence, and discomfort (e.g., cybersickness or motion sickness) sequentially (Weech et al., 2019), one must be cautious to not overload the participant with queries on different sensations (e.g., multiple vection measures and detailed questions on sickness symptoms) and states (e.g., the sense of presence).

4. It is highly recommended to offer participants a vection measure that includes the option to indicate that no vection was experienced at all. This can be done either by combining measures or by using a respective rating scale (e.g., 0–10).

5. When detailing the experimental procedure in the manuscript, we recommend refraining from paraphrasing the instructions given to participants to measure their vection and instead reporting these instructions verbatim. For example, when using a vection rating scale one might instruct the participant to “*please rate the intensity of your self-motion sensation*” whereas when one utilizes magnitude estimation the instruction might have been “*please rate the intensity of your self-motion sensation with respect to the first stimulus*”. Explicitly including these statements in a manuscript helps to clearly understand the used methodology and to interpret the results accordingly.

6. When utilizing rating scales, it is also important to avoid paraphrasing when describing the end points of the scale in the manuscript and to denote the *exact* end points of the scale and the definition of intermediate response options (if given). For example, if the left and right anchors of the scale were “*No sensation of self-motion*” and “*Very strong self-motion sensation*”, one should not detail these anchors in the manuscript as “*no vection*” and “*very strong vection*”. Moreover, it should be specified whether the scale was ordinal, numerical, or continuous.

7. Be prepared to mitigate setbacks. It is likely that participants will become overwhelmed by the sensations the vection-inducing sensory stimuli may elicit and, as such, forget to press a button or pull on a joystick. Furthermore, participants may misinterpret task instructions and respond to different aspects of the display (e.g., the velocity component). These challenges could be mitigated by presenting a practice trial, verifying that participants understood the task instructions after the practice trial, and possibly reinstructing the participant. It is also important to debrief participants, through which one could uncover *how* participants performed the task and *wha*t they experienced.

## Conclusions

The goal of the present paper was to review the scientific literature in order to provide the readership with a general overview of the most common measures utilized in vection research. A variety of different methodological approaches were identified and assigned to three categories: quantitative, chronometric, and indirect measures. For each of these measures, we discussed the benefits and limitations and provided recommendations on how to best select and use these measures when conducting empirical vection studies. Ideally, the measure(s) of choice should provide participants the option to disclose they did not experience vection, either by combining different measure types or utilizing a measure with a “null” response option. Furthermore, combining chronometric measures with quantitative response measures is advisable to capture the multidimensional aspect of vection and allow for a multivariate statistical analysis. Lastly, care should be taken not to overload participants with various measures.

## Data Availability

Data sharing is not applicable to this article as no datasets were generated or analysed during the current study.
